# 2341: Association of chronic stress with alcohol seeking and health behaviors

**DOI:** 10.1017/cts.2017.132

**Published:** 2018-05-10

**Authors:** Courtney Vaughan, Bethany Stangl, Rajita Sinha, Vijay Ramchandani

## Abstract

OBJECTIVES/SPECIFIC AIMS: The objective of this analysis was to characterize the impact of stress, both early life and chronic, on intravenous alcohol self-administration (IV-ASA) in healthy non-dependent drinkers using the Computer-Assisted Infusion System (CAIS). Personality measures also have shown to impact drinking behavior, particularly impulsivity. Few studies have assessed the impact of stress and impulsivity on drinking behaviors in a non-dependent population. METHODS/STUDY POPULATION: Healthy non-dependent drinkers (n=28) completed a CAIS session, where they push a button adlib to self-administer standardized IV alcohol infusions. Participants completed the Cumulative Chronic Stress interview and the Early Life Stress Questionnaire (ELSQ) for stress measures. The Cumulative Chronic Stress interview was broken up into 4 sections: major life events, life traumas, recent life events, and chronic stressors. The number of endorsed events was added up to create 4 separate scores. Subjective response and craving measures were collected serially using the Drug Effects Questionnaire (DEQ) and Alcohol Urge Questionnaire (AUQ). The Impaired Control Scale (ICS) assessed failed control over recent drinking in the past 6 months. Impulsivity was assessed using the NEO personality inventory, which included the N-impulsive sub-facet, as well as the UPPS-P Impulsive Behavior Scale. RESULTS/ANTICIPATED RESULTS: Results showed early life stress events (ELSQ) are related to more chronic stressors in the cumulative chronic stress interview (*p*=0.005). Participants with higher chronic stress scores showed lower subjective effects, as measured by the DEQ, following the priming exposure (*p*=0.036) but had more craving for alcohol as measured by the AUQ (*p*=0.009). A regression analysis showed the number of chronic stressful events predicted ICS failed attempts to control drinking (*p*=0.034), after covarying for sex. Participants with more chronic stressful events showed more impulsivity on the N-impulsivity measure (*p*=0.034) and the UPPS-P positive urgency measure (*p*=0.005). DISCUSSION/SIGNIFICANCE OF IMPACT: Non-dependent drinkers with more early life stress tend to have a higher number of chronic stressful events. More chronically stressful events were associated with feeling less effects of alcohol and higher craving for alcohol. Participants with more chronically stressful events also appear to have more failed attempts at controlling their drinking. Future analysis will assess for mediation and moderation of these factors. Chronically stressful events and impulsive behaviors could serve as important areas for intervention for better treatment outcomes for alcohol use disorders.

